# Theoretical analysis of wake/sleep changes in brain solute transport suggests a flow of interstitial fluid

**DOI:** 10.1186/s12987-022-00325-z

**Published:** 2022-04-13

**Authors:** John H. Thomas

**Affiliations:** Department of Mechanical Engineering, University of Rochester, Rochester, NY 14627 USA

**Keywords:** Cerebrospinal fluid, Interstitial fluid, Advection, Diffusion, Glymphatic system

## Abstract

Clearance of protein waste products from the brain is accomplished by a combination of advection and diffusion in cerebrospinal fluid (CSF) and interstitial fluid (ISF). In the glymphatic model, there is a flow of ISF in the interstitial space, and both advection and diffusion occur there. Such a flow of ISF would be slow and difficult to detect directly, and its existence has proved controversial. Waste clearance has been shown to occur mainly during sleep, during which the volume of the interstitial space increases substantially due to ISF emitted from astrocytes. Here I show that this volume increase of the interstitial space, by itself, should lead to a slight reduction of diffusive transport, due to dilution of the waste solute, but to a significant increase in flow rate and advective transport, due to lowered hydraulic resistance. Thus, a flow of ISF together with the observed volume increase of the interstitial space might provide an important mechanism contributing to the enhanced clearance during sleep.

## Introduction

Nearly all neurodegenerative diseases are linked to abnormal accumulations of metabolic waste products. For example, proteins such as amyloid-beta and tau form toxic aggregates that have been linked to Alzheimer’s disease. Removal of metabolic waste from the brain takes place within the interstitial fluid (ISF) and cerebrospinal fluid (CSF) by a combination of advection and diffusion. According to the glymphatic model [1, 2], the clearance is accomplished by an inflow of CSF along perivascular spaces (PVSs) surrounding pial and penetrating arteries which then enters into the interstitial space through gaps in the outer walls of the PVSs, mixes with ISF, and exits along PVSs surrounding venules and veins and the sheaths around nerves. While the inflow along the PVSs surrounding arteries and arterioles is well established, the possible existence of a flow within the interstitial space has proved to be controversial. A possible alternative to ISF flow is that the flow is limited to CSF along a continuous network of PVSs, while solutes are carried into these channels from the interstitial space by diffusion alone [3]. The existence of such a continuous flow path along PVSs from arterioles to veins has been suggested on the basis of tracer studies [4, 5].

Detecting a flow of ISF within the interstitial space presents a significant experimental challenge. The experiments must be done in vivo. A flow can only be detected after the introduction of tracers, but invasive procedures significantly suppress glymphatic function [6]. The details of the interstitial space are hidden deep in the brain and their size is below the current limit of resolution of noninvasive experimental techniques (e.g magnetic resonance imaging). There is, however, indirect evidence in favor of advective transport within the interstitial space. A recent application of optimal mass transport (OMT) analysis to experimental data indicates that advective transport dominates in the CSF while diffusion and advection both contribute to transport in the interstitial space [7]. Also, Ray et al. [8] show that the experimental data used to determine effective diffusion coefficients in the parenchyma are better represented if one allows for a flow of ISF in the interstitial space.

Here I present a theoretical argument in favor of the existence of a flow of ISF and advective transport in the interstitial space, based on the experimental evidence that the waste clearance occurs primarily during sleep, during which the volume of the interstitial space is increased substantially by fluid expelled from astrocytes [9].

## Wake-sleep changes in brain clearance and the extracellular space

**Fig. 1:**
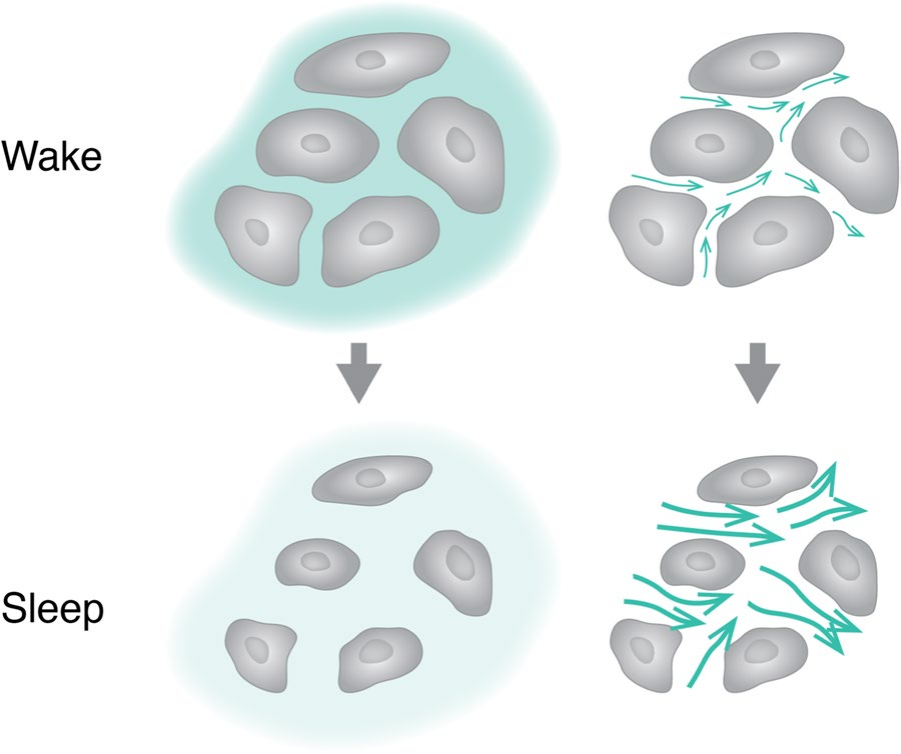
Sketch of the effect of the wake-to-sleep increase in the volume of the interstitial space (blue shading) and the porosity on the diffusive and advective fluxes of a solute. Dilution of the solute with increasing porosity decreases the diffusive flux (left panels), but reduced hydraulic resistance with increasing porosity increases the flow velocity (right panels) and the advective flux

Xie et al. [9] found that the clearance of metabolic waste products occurs primarily during sleep: there is a nearly ten-fold increase in CSF tracer influx and a doubling of amyloid-beta clearance from wake to sleep, and the volume of the interstitial space $V_{\mathrm{ISS}}$ increases by about 60% while the tortuosity $\lambda$ remains unchanged. Sherpa et al. [10] found that when a beta-adrenergic receptor agonist was applied to rat brain slices, $V_{\mathrm{{ISS}}}$ decreased significantly, due mostly to an increase in the volume of astrocytes. They suggest that norepinephrine released during the awake state decreases $V_{\mathrm{{ISS}}}$ by increasing the volume of the astrocytes following beta-adrenergic receptor activation. This suggestion is supported by several earlier studies that show that astrocytes can change their volume rapidly, while changes in neuronal cell volume are small, e.g. [11–13]. The results of these studies indicate that there is an exchange of fluid from the astrocytes to the interstitial space when transitioning from wake to sleep. A metabolic waste product, such as amyloid-beta, is produced mainly in the neurons, not in the astrocytes. Kang et al. [14] found that the interstitial concentration of amyloid-beta declines rapidly when mice transition from wake to sleep. Thus, for the analysis here we may assume that the ISF expelled from the astrocytes into the interstitial space does not contain the solute, and hence the concentration of that solute is reduced in the interstitial space in going from wake to sleep. This dilution also reduces the concentration gradient ${\varvec{\nabla }}C$ because the length scale for the gradient, which is the typical distance between penetrating arterioles, remains unchanged. Thus, diffusive transport of the solute is reduced in going from wake to sleep. On the other hand, assuming there is a flow of ISF, the increased volume of the interstitial space reduces the hydraulic resistance and thus increases the flow rate and the advective transport. These two processes are illustrated schematically in Fig. 1. The magnitude of the changes in flux rates can be estimated by means of a scale analysis, presented in the following section.

## Scale analysis of wake-sleep changes in the diffusive and advective fluxes

Consider a control volume $V_{\mathrm{{TOT}}}$ of the interstitium. The volume $V_{\mathrm{{ISS}}}$ of the interstitial space within this control volume can be expressed in dimensionless form as the *porosity* $\alpha = V_{\mathrm{{ISS}}}/V_{\mathrm{TOT}}$. In going from wake to sleep, $\alpha$ increases. Assume that this increase in $\alpha$ is due to ISF expelled mainly from astrocytes (not neurons) and hence does not add more solute (amyloid-beta). Then the total mass of solute in the interstitial space remains unchanged, and the concentration *C* in the interstitial space scales as $C \sim C_0/\alpha$, where $C_0$ is a constant reference concentration (the value for $\alpha = 1$), and hence *C* decreases from wake to sleep due to dilution.

The diffusive flux rate (transport per unit area per unit time) is given by Fick’s first law, $\mathbf{F} _{\mathrm{D}} = -D^* {\varvec{\nabla }}C$, where $D^* = D/\lambda ^2$ is the effective diffusivity and *D* is the free diffusivity. The concentration gradient ${\varvec{\nabla }}C$ scales as *C*/*L*, where *L* is the typical distance between arterioles and venules. This distance *L* does not change from wake to sleep, so the concentration gradient scales as $C_0/\alpha L$ and therefore is decreased by the dilution of *C* in going from wake to sleep. The diffusive flux then scales as

1 $$\begin{aligned} |\mathbf{F} _{\mathrm{D}}| \sim D^* \frac{C}{L} \sim \left( \frac{D C_0}{\lambda ^2 L} \right) \frac{1}{\alpha } . \end{aligned}$$

If we assume that the tortuosity $\lambda$ is unchanged, then all the terms in parentheses in the last expression are constant and hence the diffusive flux rate scales as $1/\alpha$. The net transport of solute per unit time across a surface *S* with unit normal vector $\mathbf{n}$ is

2 $$\begin{aligned} \mathcal {F_{\mathrm{D}}} = \int _S \mathbf{F} _{\mathrm{D}} \cdot \mathbf{n} dS , \end{aligned}$$

and here we must account for the increase in the portion of the surface *S* over which the flux actually occurs, which increases as the porosity increases. (Here *S* may be considered as the outer surface of the PVS into which the solute is diffusing.) This portion of *S* scales as the cross-sectional area of the interstitial space, and hence as $\alpha ^{2/3}$. Hence, according to equations (1) and (2), ${\mathcal {F}}_{\mathrm{D}}$ scales as

3 $$\begin{aligned} \mathcal {F_{\mathrm{D}}} \sim \left( \frac{D C_0}{\lambda ^2 L} \right) \frac{1}{\alpha ^{1/3}} . \end{aligned}$$

Thus, in going from wake to sleep, as $\alpha$ increases the diffusive flux of solute out of the interstitial space is decreased. If diffusion is the dominant mechanism for solute removal from the interstitial space, then this result is difficult to reconcile with the observed increase in clearance during sleep.

Now let us consider the effect of increased porosity on the advective flux rate of the solute due to a presumed flow of ISF in the interstitial space. The advective flux rate is $\mathbf{F} _{\mathrm{A}} = - C \mathbf{u}$, where $\mathbf{u}$ is the fluid velocity. For flow in the interstitial space we adopt the Darcy law for flow in a porous medium, driven by a pressure gradient ${\varvec{\nabla }}p$:

4 $$\begin{aligned} \mathbf{q} = -\frac{\kappa }{\mu } {\varvec{\nabla }}p , \end{aligned}$$

where *p* is the pressure, $\kappa$ is the permeability, $\mu$ is the dynamic viscosity, and $\mathbf{q}$ is the superficial (Darcy) velocity, related to the actual velocity by $\mathbf{u} = \mathbf{q} / \alpha$. The permeability $\kappa$ is given in terms of the porosity $\alpha$ and tortuosity $\lambda$ by the Kozeny-Carman equation,

5 $$\begin{aligned} \kappa = \frac{\alpha ^3}{\lambda {\mathcal {A}}^2 (1 - \alpha ^2)} , \end{aligned}$$

where ${\mathcal {A}}$ is the specific surface area. This gives the scaling:

6 $$\begin{aligned} |\mathbf{F} _{\mathrm{A}}| \sim \frac{C_0}{\alpha } \frac{\kappa |{\varvec{\nabla }}p| }{\alpha \mu } \sim \frac{C_0 |{\varvec{\nabla }}p|}{\alpha ^2 \mu } \frac{\alpha ^3}{\lambda (1 - \alpha ^2){\mathcal {A}}^2} \sim \left( \frac{C_0 |{\varvec{\nabla }}p|}{\lambda \mu {\mathcal {A}}^2} \right) \frac{\alpha }{(1 - \alpha ^2)}. \end{aligned}$$

The net transport of solute by advection per unit time across a surface *S* is

7 $$\begin{aligned} \mathcal {F_{\mathrm{A}}} = \int _S \mathbf{F} _{\mathrm{A}} \cdot \mathbf{n} dS , \end{aligned}$$

Again, we must account for the increase in the portion of the surface *S* over which the flux actually occurs, which increases as $\alpha ^{2/3}$ as the porosity increases. The scaling for ${\mathcal {F}}_{\mathrm{A}}$ is thus

8 $$\begin{aligned} \mathcal {F_{\mathrm{A}}} \sim \left( \frac{C_0 |{\varvec{\nabla }}p|}{\lambda \mu {\mathcal {A}}^2} \right) \frac{\alpha ^{5/3}}{(1 - \alpha ^2)} . \end{aligned}$$

If we assume that the tortuosity $\lambda$, specific surface area ${\mathcal {A}}$, and pressure gradient ${\varvec{\nabla }}p$ are unchanged, then the terms in large parentheses in expressions (6) and (8) are constant, so ${\mathcal {F}}_{\mathrm{A}} \sim \alpha ^{5/3}/(1- \alpha ^2)$, and hence the advective transport increases from wake to sleep.

**Fig. 2:**
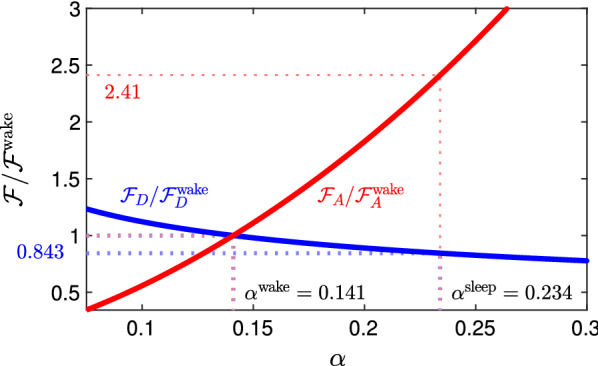
Scaling of the diffusive and advective flux rates in the interstitial space, $\mathcal {F_{\mathrm{D}}}$ and $\mathcal {F_{\mathrm{A}}}$, with changes in porosity $\alpha$. In each case, the flux is normalized by its (unknown) value in the awake state. The values of the porosity for wake ($\alpha = 0.141$) and sleep ($\alpha = 0.234$) are those determined by Xie at al. for the mouse brain. In going from wake to sleep, the diffusive flux decreases slightly while the advective flux increases by a factor of nearly 2.5

Fig. 2 shows plots of the scaling relations for the diffusive flux rate $\mathcal {F_{\mathrm{D}}}$ (eq. 3) and the advective flux rate $\mathcal {F_{\mathrm{A}}}$ (eq. 8) as functions of the porosity $\alpha$. In each case, the flux is normalized by its unknown value in the awake state, so the relative change is depicted. The indicated values of the porosity in the wake and sleep states, $\alpha$ = 0.141 and 0.234, are those found by Xie et al. [9] for the mouse brain. We see that the diffusive flux rate decreases slightly in going from wake to sleep, while the advective flux rate is increased substantially, by a factor of 2.4. Interestingly, an increase by a factor of about 2.4, from wake to sleep, in the clearance rates of injected amyloid-beta and an inert tracer was reported by Xie at al. [9]. Also, Jin et al. [15] developed a model of combined advection and diffusion in a geometric model of the interstitium, in which they solved the Navier-Stokes equation rather than using the Darcy equation for a porous medium. They found that the flux rate of tracers entering the interstitial space increased by a factor of very nearly 2 when they increased the volume fraction from $\alpha = 0.1$ to $\alpha = 0.24$. This is in good agreement with the finding here that the flux rate increases by a factor of 2.4 in going from $\alpha = 0.14$ to $\alpha = 0.234$, especially when we consider that they included diffusion in their calculation which, according to the scaling arguments above, would act to slightly offset the increase in flux rate caused by increased advection. This agreement also helps to justify the use of the Darcy equation to represent the flow in the interstitium.

Xie et al. [9] found that there was no significant change in tortuosity between wake and sleep, and accordingly the scale analysis presented above assumes constant tortuosity. Theoretical models support this assumption [16]. Tortuosity is in general a complex parameter involving several geometric effects, but for simple models the tortuosity can be expressed as a function of the porosity alone: for example, we have the expression derived by Maxwell for a dilute suspension of solid spheres,

9 $$\begin{aligned} \lambda = \sqrt{\frac{3-\alpha }{2}} . \end{aligned}$$

Based on this formula, the increase in $\alpha$ from 0.141 to 0.234 observed by Xie et al. corresponds to only a very slight decrease in $\lambda$, from 1.196 to 1.176. Even if we were to allow changes in tortuosity and use the Maxwell formula (9) for $\lambda$ in the scale analysis above, all of the principal conclusions still hold.

## Discussion

Here I have examined the effect of just one of several possible wake-sleep changes on waste clearance from the brain: the increase in porosity of the interstitium. Other changes might also affect the clearance rate. For example, an increase in the size of PVSs during sleep would lower their hydraulic resistance and thus promote a greater overall flow rate along them. Perhaps the driving mechanism for PVS flow is stronger during sleep. However, increasing the flow of CSF along PVSs will not, by itself, enhance overall clearance significantly if diffusion is the only transport process within the interstitial space. A faster flow along the PVSs might establish a slightly lower concentration within them, thereby slightly increasing the concentration gradients driving diffusion in the interstitial space, but this slow diffusive process will still govern the overall clearance rate, and the ability to speed up clearance in this way is quite limited. On the other hand, a large wake-sleep increase in clearance rate would be much more easily generated by the significant increase in flow rate in the interstitial space that would occur because of increased porosity and lowered hydraulic resistance there.

The hydraulic resistance of the interstitial space is inversely proportional to its permeabilty, which is a poorly known quantity: estimates of its value in the mouse brain span more than two orders of magnitude [17, 18], and the low value of permeability (high resistance) has been the basis for arguments against any significant flow of ISF [15, 18]. However, Ray et al. [8] find a better fit to the experimental data used to determine effective diffusion coefficients in the parenchyma if one includes a flow of ISF in the interstitial space, with a typical flow speed of 50 $\mu$m/min. At such a flow speed, advection is the dominant transport mechanism for large molecules in the interstitial space (Péclet number greater than unity). Also, Koundal et al. [7] applied optimal mass transport analysis to DCE-MRI images of tracer distribution in live rat brains and found that both advection and diffusion were required to match the observed tracer patterns in the interstitium. A computational model of flow in the interstitium shows that the hydraulic resistance depends on the relative numbers of arterioles and venules and their spatial arrangement, both of which vary among species [19]. In future work, the dependence of glymphatic flow on the hydraulic resistance of the interstitium can be examined with the aid of a recently developed global hydraulic network model [20]. Although the significance of a flow of ISF in the brain parenchyma is still an open question, I have shown here that such a flow would go a long way toward explaining the established wake-sleep variation in waste clearance from the brain.

## Data Availability

Not applicable.
