# Correction: Theoretical analysis of wake/sleep changes in brain solute transport suggests a flow of interstitial fluid

**DOI:** 10.1186/s12987-025-00705-1

**Published:** 2025-10-08

**Authors:** John H. Thomas

**Affiliations:** Department of Mechanical Engineering, University of Rochester, Rochester, NY 14627 USA

**Correction: Fluids and Barriers of the CNS (2022) 19:30** 10.1186/s12987-022-00325-z

In this article [1], an incorrect expression of the Kozeny-Carman equation was used. The expression for the permeability $\:\kappa\:\:$ in Eq. (5) should read

1 $$ \kappa ~ = \frac{{\alpha ^{3} }}{{\uplambda {\mathcal{A}}^{2} \left( {1 - \alpha } \right)^{2} }}, $$

and the corresponding expression for the scaling of the advective flux rate (eq. 8) should read

2 $$ {\mathcal{F}}_{A} \sim \left( {\frac{{C_{0} \left| {\nabla _{p} } \right|}}{{\uplambda \upmu \mathcal{A}^{2} }}} \right)\frac{{\alpha ^{{{5 \mathord{\left/ {\vphantom {5 3}} \right. \kern-\nulldelimiterspace} 3}}} }}{{\left( {1 - \alpha } \right)^{2} }}. $$

A corrected version of Fig. 2 is presented here, showing plots of the normalized scaling relations for the diffusive flux rate ${\mathcal{F}}_{\text{D}}$ and the advective flux rate ${\mathcal{F}}_{\text{A}}$ as functions of the porosity α.

The curve for $\mathcal{F}_{\text{A}} |/\mathcal{F}_{\text{A}}^{{{\text{wake}}}}$ is slightly steeper than that in the original figure, leading to a somewhat greater value at the sleep state (2.93 versus 2.41). This slightly greater increase in the flow rate and advective transport, due to lowered hydraulic resistance, only reinforces the main conclusions drawn in this paper.

**Fig. 2:**
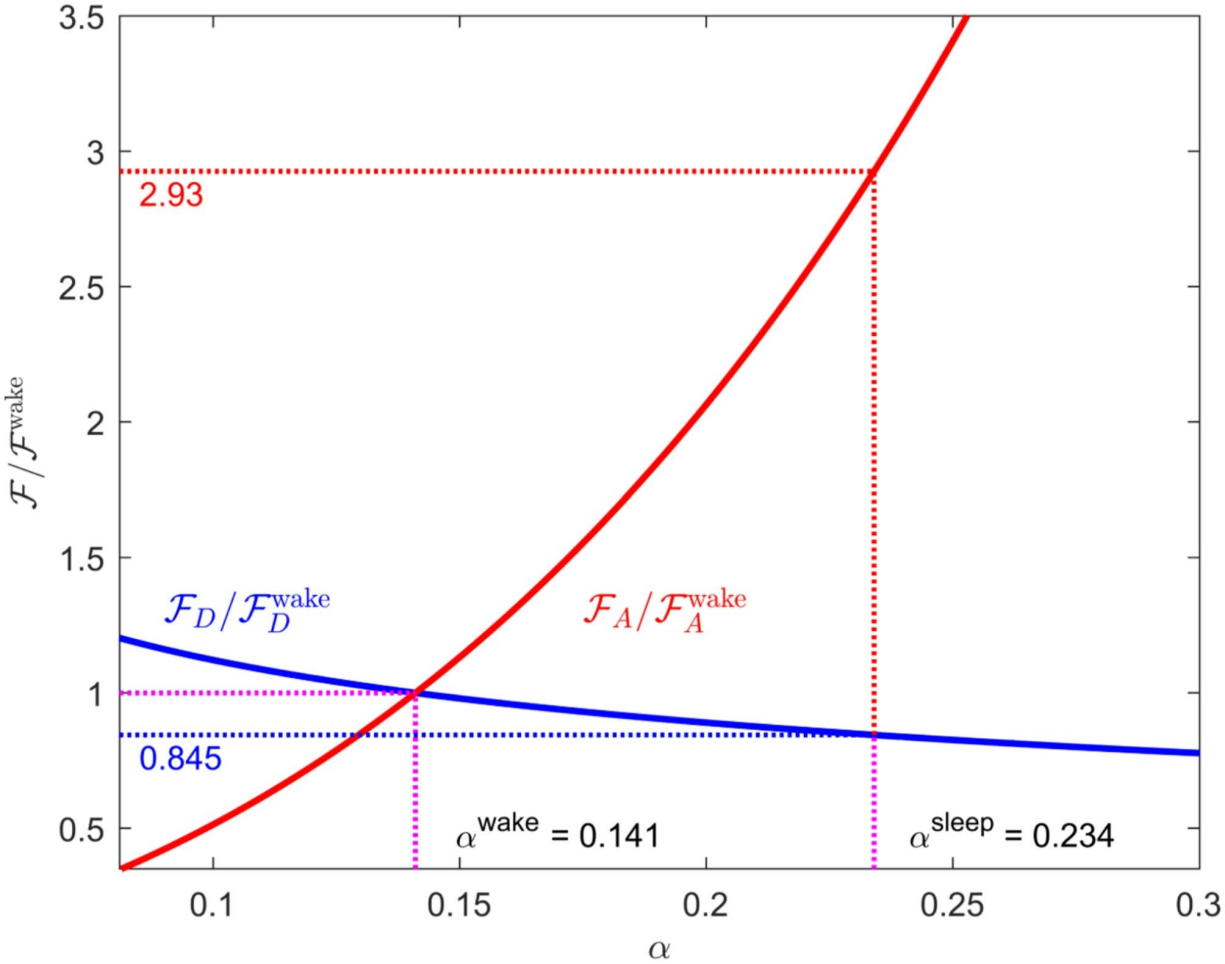
Scaling of the diffusive and advective flux rates in the interstitial space, ${\mathcal{F}}_{\text{D}}$ and ${\mathcal{F}}_{\text{A}}$ , with changes in porosity *α*. In each case, the flux is normalized by its (unknown) value in the awake state. The values of the porosity for wake (*α* = 0.141) and sleep (*α* = 0.234) are those determined by Xie at al. for the mouse brain. In going from wake to sleep, the diffusive flux decreases slightly while the advective flux increases by a factor of nearly 3

The original article has been corrected.
